# The Absence of Esprit De Corps

**Published:** 1918-04-13

**Authors:** 


### NURSING AFFAIRS IN IRELAND.

### The Absence of Esprit de Corps.

Esprit de corps and union are twin brethren. They are even more closely allied, for without the one the other is impossible. In the medical profession and in the legal, *esprit de corps* is a predominant quality. I do not know how it arrives—perhaps somebody may explain—but it is there, and it permeates all ranks. To quote Scripture, when one member suffers, all the members suffer with it. The phenomenon is worth studying, because although loyalty to the common cause is general, individual professional jealousy is by no means absent. Doctors and lawyers are quite as human as other people, and they vie with each other in their practice just as tradespeople do in their anxiety to sell their wares. That they should vigorously, not to say fiercely, resent any intrusion on their domain, or any invasion of their professional rights, is something that courts admiration. Doctors and lawyers have their unions, but whether *esprit de corps* precedes or succeeds union I am unable to say. I can only point out that they are allied forces, each dependent on the other for its existence.

### An Absence of Esprit de Corps.

I regret to have to chronicle that in the nursing profession, in Ireland at all events, *esprit de corps* is conspicuous by its absence. Hence, as a natural consequence, union is absent. I am convinced that the man-in-the-street (or, otherwise, the lay public) pay far more court to the nurse's uniform than nurses themselves pay. I am offering a personal opinion; but before stating it I took the opportunity of consulting doctors and nurses of eminence, and their opinion is pronounced and emphatic that this is a fact. Nurses, they say, are loyal to their hospital while they are there, and they are loyal to each other seldom or never. Personal interest overrides every other consideration, with the inevitable result that the common interests of the profession are submerged. I venture to submit these suggestions to the leaders of the nursing profession. I entertain no doubt that there are many exceptions, and the more the better, but if it is true in general that *esprit de corps* is lacking some effort ought to be made to inculcate that which is in reality a fundamental essential to prosperity. I am dealing not with sentiment, but with a bread-and-butter question. The lay public are behind the nurses, but if the nurses fail to appreciate the fact they will miss the tide, and this they can easily miss by pursuing a policy of individualism. Matrons and doctors in hospitals where nurses are trained might do a great deal to foster a spirit of comradeship. A timely word might be spoken at every lecture, and the matter treated as a duty, which it really is.

### The Economic Problem in Ireland.

I am convinced that in Ireland especially the leaders of nurses will never accomplish anything very useful unless they face the economic problem with boldness and decision. The task may be delicate and difficult, but there is no evading the issue that a battle must be fought with the hospitals, and, of course, matrons naturally shrink from this. The hospitals are poor, many of them almost bankrupt, dragging along in the hope of a windfall of some sort, and the services of the nurse and the fees of probationers are regarded as a substantial source of revenue. Instead of being in the position of a woman pursuing a career she is an involuntary philanthropist. The physician and the surgeon work free at hospitals, but the circumstances are entirely different. They are of untold value to the poor, as are the hospitals themselves. But they are acquiring valuable experience which afterwards makes for their success as practitioners. The voluntary service of the medical profession in charity hospitals is not sacrifice: the nurse's work is nothing else. This is neither fair nor just no matter how hard the struggle of the hospital may be. The hospital charwoman and cook receive the normal wages of their class. The grocer and butcher sell to hospitals at market prices. Why should the nurse be the sole victim in the case? Here are in brief the conditions of service—robust health and good character; probation fee, from fifteen to fifty guineas; four years' free service; salary thereafter from £10 to £25. Thus, for seven years, a nurse, who begins at the average age of twenty-three, works in a hospital at about half the wages of a general domestic servant.

### Union and Better Pay.

My contention is that if a vigorous propaganda is instituted with the object of securing a union of nurses there must be, on the part of the College of Nursing in Ireland, an equally vigorous effort made to raise the remuneration scale. Otherwise nurses will fail to see for what purpose they are expected to pay their guineas. I entertain no doubt that the public will support the legitimate claims of the nurse, but the leaders will find it necessary to advocate the cause, and to press the case on the attention of the governors of hospitals. Whether this would have the effect of producing *esprit de corps* or not remains to be seen. It would certainly tend to union. IERNE.

